# Salary delays among public sector primary care workers: evidence from facility surveys across 16 low- and middle-income countries

**DOI:** 10.1136/bmjgh-2024-017742

**Published:** 2025-12-11

**Authors:** Han Zhang, Mansha Mahajan, Kevin Croke, Sebastian Bauhoff, Peter Waiswa, Margaret McConnell

**Affiliations:** 1 Department of Global Health and Population, Harvard T H Chan School of Public Health, Boston, Massachusetts, USA; 2 School of Public Health, Makerere University, Kampala, Uganda; 3 Makere University Maternal and Newborn Center of Excellence, Kampala, Uganda

**Keywords:** Global Health, Health policy, Health systems, Public Health

## Abstract

**Background:**

Primary healthcare is crucial for universal health coverage in low- and middle-income countries. While research on improving workforce performance has focused on training and incentives, the impact of basic payment system functions remains underexplored. This study investigates salary delays among public-sector primary care workers across 16 low- and middle-income countries and the association of delays with worker outcomes.

**Methods:**

We analysed data from World Bank Service Delivery Indicators and Health Results-Based Financing surveys (2010–2018), covering 22 003 primary care workers from 8301 public-sector facilities. Salary delay was defined as any self-reported delay in receiving the previous month’s base salary. We examined patterns of delays and their associations with worker motivation, satisfaction and performance using fixed-effects linear probability models.

**Findings:**

On average, 37% of health workers experienced salary delays, ranging from 2% to 83% across countries, primarily due to funding shortages and administrative issues. Delays were more common among workers without formal contracts and in rural or lower-level facilities. Experiencing a salary delay was associated with a 3.1 percentage point (pp) lower probability of being satisfied with one’s salary (95% CI −5.7 to −0.5), a 4.6 pp lower probability of feeling motivated (95% CI −7.0 to −2.3), a 1.9 pp higher probability of unauthorised absence (95% CI +0.3 to +3.5) and a 5.6 pp higher probability of outside employment (95% CI +2.5 to +8.6).

**Interpretation:**

Salary delays are prevalent among public primary healthcare workers in resource-poor settings, affecting vulnerable groups and associated with negative worker outcomes. Addressing delays requires diagnosing bottlenecks across administrative tiers, improving cash-to-payroll execution and allocating resources equitably to settings and worker groups where delays are most concentrated. Future work should examine payment processes across administrative levels and incorporate routine measurement of salary timeliness to support accountability and inform targeted strategies to reduce delays.

**WHAT IS ALREADY KNOWN ON THIS TOPIC**

- Health workers are essential to primary care delivery in low- and middle-income countries (LMICs), yet workforce performance challenges persist. While training and financial incentives have been extensively studied, payment timeliness has been seldom examined quantitatively. Qualitative evidence suggests that payment delays can undermine worker motivation, service quality and programme effectiveness, yet multi-country data on the prevalence, patterns, determinants and consequences of salary delays are scarce.

**WHAT THIS STUDY ADDS**

- This study provides the first multi-country quantitative analysis of salary delays among public-sector primary care workers using facility survey data from 16 LMICs. It highlights that base salaries, often assumed to be reliable, are frequently delayed due to funding shortages and administrative inefficiencies, disproportionately affecting workers without formal contracts and in rural or lower-level facilities, and are associated with lower motivation and job satisfaction, increased absenteeism and reliance on outside employment.

**HOW THIS STUDY MIGHT AFFECT RESEARCH, PRACTICE OR POLICY**

- These findings underscore the urgency for policymakers to improve healthcare financing and cash-to-payroll execution in the public health sector of LMICs and to ensure equitable resource allocation to settings and worker groups where delays are concentrated. Routine measurement of salary timeliness, paired with documentation of payment processes across administrative levels, can support accountability and guide targeted reforms.

## Introduction

 Primary healthcare (PHC) is a vital part of health systems and can help achieve universal health coverage by promoting population health, reducing preventable deaths, addressing growing burdens of chronic disease, managing infectious epidemics and providing accessible, equitable, quality care.^1 2^ In low- and middle-income countries (LMICs), 55% of health spending goes towards PHC, with a large share allocated to workforce wages.^3^ Public sector workers constitute the majority of the PHC workforce in most countries, serving as the first point of patient contact, especially in remote and rural areas.^4^,^7^ However, many LMICs face challenges retaining PHC workers, with an estimated 15 million health worker shortfall by 2030 concentrated in these settings.^8^ The existing PHC workforce often reports poor job satisfaction, and previous research has documented behaviours such as absenteeism, moonlighting and informal fee-taking, undermining quality of care and patient outcomes.^9^,^13^ Maintaining a well-functioning public-sector PHC workforce is critical for effective PHC systems.^14 15^

Existing research on factors influencing health worker motivation, satisfaction, retention and performance has focused on training/education, improving facilities and offering incentives.^14^ However, evidence to date suggests that these strategies alone cannot fully address challenges faced by the public PHC workforce in LMICs.^7 14 16^ Provider training, facility upgrades and improved supervision have had mixed, modest or short-term impacts on patient outcomes.^14 17 18^ Moreover, PHC workers may not adhere to guidelines despite demonstrating appropriate knowledge, and even targeted performance-based incentives have limited impacts on provider effort and quality of care.^1719^,^21^

Core payment system functions for PHC workers have received less attention. Timely, complete payment of base salaries is a basic function of public financial management (PFM),^22 23^ including budget credibility, in-year cash releases, commitment control, payroll administration and last-mile disbursement.^22 23^ When these functions falter, payment uncertainty can undermine motivation and blunt the effects of downstream quality and performance reforms.^24 25^ This gap is particularly concerning in LMICs, where many workers face financial insecurity and rely on prompt and complete payment to fulfil immediate needs. Assessing payment functions is further complicated by scarce and inconsistent payment data, complex pay structures and worker surveys with limited samples.^26^,^28^ While prior studies have documented the amounts and composition of health workforce payment in parts of Africa, payment processes and timeliness remain underexplored.^26^,^28^ Understanding salary timeliness is therefore critical for identifying constraints on worker motivation, satisfaction and performance and for informing practical improvements in payment execution and, ultimately, care delivery effectiveness.

In this paper, we investigated the prevalence of delays in base salary payments for health workers in LMICs. Using detailed health facility survey data across 16 LMICs between 2010 and 2018, we analysed patterns of salary delays among PHC workers by individual and facility characteristics, as well as the underlying reasons and duration of these delays. We also examined the associations between salary delays and critical aspects of health worker well-being and behaviour, including motivation, job satisfaction, absenteeism and outside employment.

## Methods

### Data sources

We compiled a dataset of health workers using large-scale facility surveys from the World Bank’s Service Delivery Indicators (SDI) project^29^ and Health Results-Based Financing (RBF) Impact Evaluation programme^30^ across 16 LMICs between 2010 and 2018. These surveys provided individual-level data on health worker cadre, educational attainment, payment attributes, performance and other basic personal and facility characteristics. The RBF surveys additionally collected details on workers’ contract type, payment incentive structures, job satisfaction and motivation. The SDI health surveys used a nationally representative two-stage stratified random sampling design, whereas RBF surveys sampled all facilities within selected regions. For the health worker interviews, RBF interviewed all health workers present at a facility on the survey day, whereas SDI surveyed a random subset of workers identified from a staff roster obtained during an initial visit during unannounced follow-up visits. Workers who were absent during the follow-up visits were not included. Both surveys used standardised questionnaires and data collection protocols, providing data that are comparable across countries and years within each survey. Online supplemental appendix table A1 provides full details on the sampling approach, geography, facility and worker coverage.

### Measures

**Figure 1:**
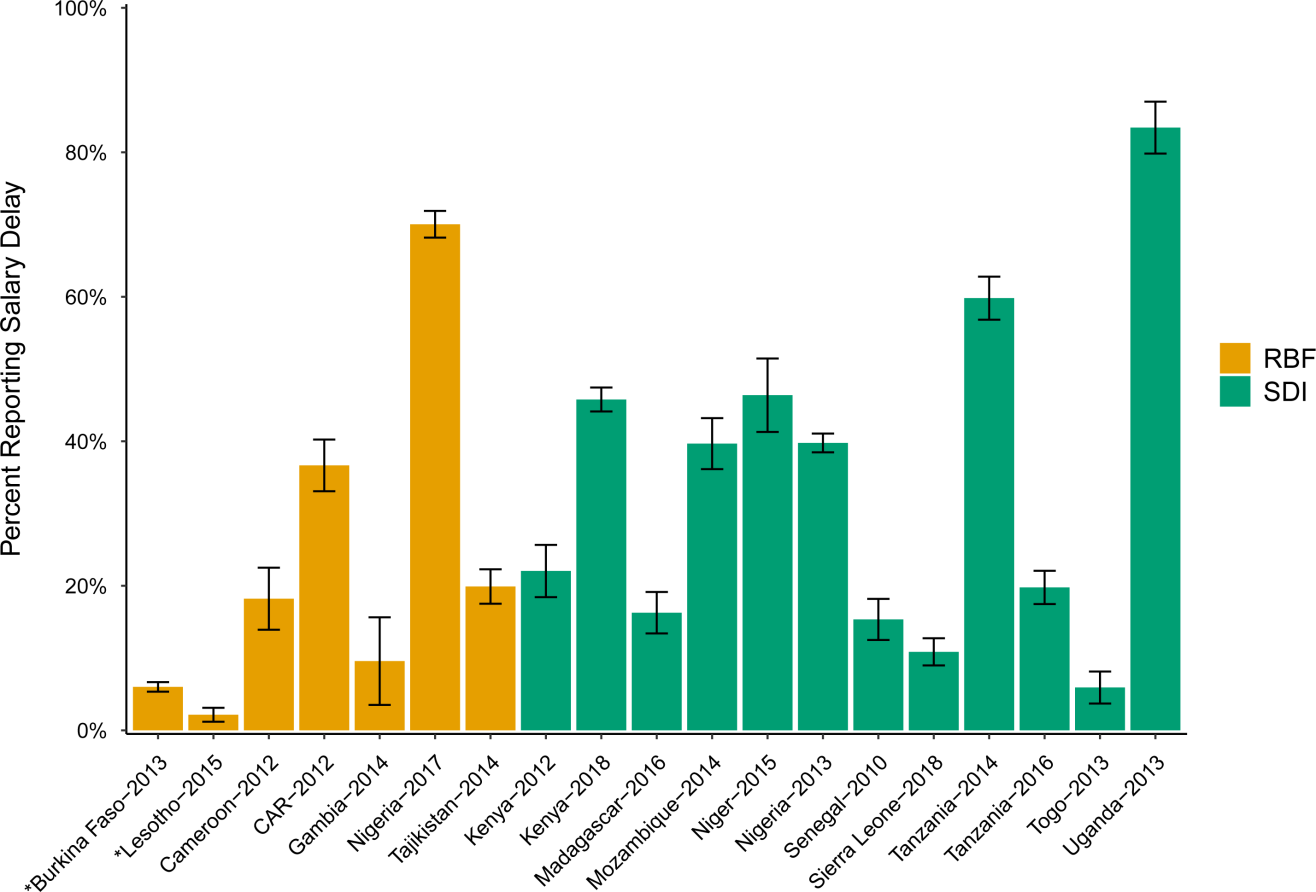
Salary delay among public-sector PHC workers in 16 LMICs. Notes: Salary delay is defined as any delay in receiving the previous month’s base salary beyond the scheduled pay date, self-reported by health workers; delays in other compensation types (allowances/bonuses) and changes in contractual pay frequency were excluded. A random sample (SDI surveys) or all (RBF surveys) of the clinical health workers present were asked whether they had experienced a delay in receiving their full base monthly salary for the preceding month. For comparability, bars for the two longer-recall waves (Burkina Faso: 3 months; Lesotho: 12 months) display a recall-standardised lower-bound previous-month rate (reported prevalence ÷ 3 or ÷ 12). Because delays can persist across months for the same worker, these values likely underestimate true previous-month prevalence. Original longer-recall prevalences (unscaled): Burkina Faso, 18.0% (95% CI 16.0% to 20.0%); Lesotho, 25.9% (95% CI 14.2% to 37.5%). Error bars indicate 95% CIs for the wave-specific prevalence. *Longer recall (lower-bound previous-month rate). LMICs, low- and middle-income countries; PHC, primary healthcare; RBF, results-based financing; SDI, Service Delivery Indicators.

The primary outcome was salary delay, defined as any self-reported delay by a health worker in receiving last month’s base salary payment beyond the scheduled pay date. Delays in non-salary compensation such as incentive, allowances or benefits were excluded, as were delays related to changes in payment frequency like contractual alterations. In the facility surveys, a random sample (SDI) or all present clinical health workers (RBF) were asked whether they had experienced a delay in receiving their full base monthly salary for the preceding month. Two survey rounds used a longer recall period: Burkina Faso (3 months) and Lesotho (12 months). For cross-country comparability, we plotted a recall-standardised lower-bound previous-month rate (reported value ÷3 or value ÷12) and flagged them in figure 1. Because salary delays can persist across months for the same worker, this transformation likely underestimates previous-month prevalence; we therefore interpreted the flagged points cautiously and provided the original recall prevalence (with 95% CIs) in the figure 1 note. In regression analysis, country-year fixed effects were introduced to account for measurement differences across countries and years.

The reasons for salary delays were assessed in the RBF surveys by asking health workers an open-ended question about typical reasons for not receiving their full salary on time in the last 12 months. Responses were coded by enumerators into predefined categories including lack of funds, systemic/administrative delays, salary withheld due to debts, performance/absence issues, unclassified or unexplained. Typically, one main reason was recorded per worker, but multiple reasons were recorded in two survey rounds if cited by the respondent. Length of salary delay was captured by asking how many days the previous month’s salary was delayed, coded into a binary variable indicating if the delay extended beyond 1 month since the question only recorded delays up to 31 days or no payment received yet.

Outcomes on health worker motivation, job satisfaction, unauthorised absenteeism and outside employment were constructed and harmonised across surveys for comparability. Online supplemental appendix table A2 details the measurement variations and harmonisation approaches. Motivation and satisfaction were specifically measured using the validated Minnesota Satisfaction Questionnaire and Job Satisfaction Survey instruments.^31 32^ Health workers were asked to rate their subjective motivation, overall job satisfaction and salary satisfaction on 4- or 5-point Likert scales (eg, 1=strongly disagree, 5=strongly agree) or frequency rating scales (eg, 1=rarely, 5=frequently). To enhance comparability, ordinal responses were converted into binary indicators of whether workers reported being motivated, satisfied with their job or satisfied with their salary more than half of the time (ie, a rating above the scale midpoint of 2 or 3). This dichotomisation approach is for simplified interpretation and is guided by the psychological well-being measurement literature for distinguishing between high and low motivation/satisfaction.^33^

We measured disruptive behaviours including unauthorised absenteeism and outside employment. In RBF surveys, workers self-reported whether they had ever been absent from work without authorised leave. In the SDI surveys, facility supervisors directly reported and verified any unauthorised absences by workers. An absence was considered unauthorised if not approved. Outside employment was measured by whether workers had any other job or income-generating activity to supplement their earnings besides their primary facility role.

### Study sample

We included all health facility surveys with at least one question on health worker payment, resulting in a sample of 16 countries: Burkina Faso, Central African Republic (CAR), Cameroon, Gambia, Kenya, Lesotho, Madagascar, Mozambique, Niger, Nigeria, Sierra Leone, Senegal, Tajikistan, Tanzania, Togo and Uganda. Despite a general trend towards health system decentralisation in these countries, variations exist in administrative structures and subnational responsibilities, including oversight of funds and personnel management. Online supplemental appendix table A3 provides an overview of these structures to contextualise our analyses.

We focused on the health workers providing primary care (eg, maternal and child health services, vaccination) at public-sector facilities. We restricted the analytic sample to public-sector facilities, where salaries are typically set centrally and paid through civil-service payrolls,^3^ maximising comparability across SDI and RBF rounds; by contrast, private-sector sampling frames and pay structures vary across countries and are not consistently captured in the RBF rounds, limiting comparability for our pooled design. The sample includes both facility-based workers and community health workers, the primary target of both surveys. We excluded 67 non-patient-facing staff (eg, lab technicians, administrative staff) due to differences in their payment levels and structures compared with frontline health workers (see online supplemental appendix table A4 for details). An additional 218 workers were dropped due to unclear documentation of facility details. This yielded a primary sample of 22 003 health workers from 8301 public-sector health facilities across 16 countries between 2010 and 2018.

We constructed an ‘RBF subsample’ of 4183 health workers from five RBF surveys with complete data on motivation, satisfaction, absenteeism and outside employment for additional analysis of associations between salary delays and workers’ behaviours. See online supplemental appendix figure A1 for the sample selection process and online supplemental appendix tables A5 and A6 for sample composition.

### Analytical approach

We followed an exploratory approach to examining salary delays among the public-sector PHC workforce. We first described the demographic composition of the PHC workforce and examined the frequency of reported salary delays in the primary sample. To identify where delays were concentrated, we analysed delay patterns by worker and facility characteristics using a linear probability model. We adjusted for country, year and data source fixed effects for any measurement inconsistencies across survey rounds. Model specifications were detailed in online supplemental appendix 2.1.1.

We further explored the associations between salary delays and health worker behaviour using the RBF subsample. Using a two-way fixed effects linear probability model, we examined the relationships between salary delays and behavioural indicators including satisfaction, motivation, unauthorised absenteeism and outside employment. We included interview-country and year fixed effects to account for unobserved confounding factors invariant across countries and time. We clustered standard errors at the facility level to account for multiple health workers within the same facility. Additionally, we adjusted for worker characteristics such as cadre (doctor/clinical officer, nurse/midwives, other health workers), employment contract type (permanent, fixed-/short-term, informal/volunteer/other), gender, age, rural/urban location and facility level (hospital, health centre, health post). Model specifications are detailed in online supplemental appendix 2.1.2. Estimated mean differences (MDs) from the linear probability models are absolute differences in probability on a 0–1 scale; multiply by 100 to interpret as pp.

To document data availability and transparency, online supplemental appendix table A7 summarises, for each country-year survey wave, whether each indicator was collected and the percent missing when collected. We addressed missing values in covariates using multiple imputation with chained equations and logistic regression models (details in online supplemental appendix appendix 2.2). As a sensitivity analysis, we employed the missing indicator method to assess the robustness of our results to the imputation strategy. We also conducted a leave-one-out analysis by excluding one country at a time to ensure that the pooled results were not driven by a single country.

### Patient and public involvement

Patients or the public were not involved in the design, conduct, reporting or dissemination plans of our research.

## Results

### PHC workforce composition and salary delay

The primary sample covered 22 003 public PHC workers from 8301 health facilities across 16 countries between 2010 and 2018, spanning 7 RBF and 12 SDI facility surveys (online supplemental appendix table A5). 56% of the PHC workers are from health centres, 31% from health posts serving remote and rural areas and 13% from hospitals/polyclinics.

Table 1 provides the demographic characteristics of our PHC worker sample. Females comprised the majority of the workforce (60%), though less so in CAR (33%), Gambia (31%) and Mozambique (48%). In terms of age, 38% of workers were 25–34 years old, 29% were 35–44 years old, and 28% were 45 or older. Most workers (92%) had at least a secondary education, with Niger (20%) and Togo (31%) being exceptions where significant shares of workers had less than a secondary education. The workforce consists of 12% doctors or clinical officers, 42% nurses or midwives and 46% non-clinical health workers such as community health workers or outreach vaccine workers.

**Table 1:** Demographic characteristics of public-sector PHC workers in 16 LMICs

Country-year	Female (%)	Age (%)				Education (%)			Cadre (%)		
		<25	25–34	35–44	≥45	None or primary	Secondary	Post secondary	Doctor	Nurse	Other
RBF											
Burkina Faso-2013	50.5	1.0	53.1	41.4	4.5	2.5	89.7	7.8	1.1	73.4	25.5
CAR-2012	33.2	1.9	21.7	42.2	34.2	18.6	68.1	13.3	6.1	5.0	88.9
Cameroon-2012	66.7	6.3	33.5	34.2	26.1	14.4	79.7	5.8	2.4	1.7	95.9
Gambia-2014	30.9	19.1	55.3	20.2	5.3	0.0	20.2	79.8	3.2	68.1	28.7
Lesotho-2015	91.4	1.9	39.6	20.8	37.7	0.0	1.8	98.2	0.0	100.0	0.0
Nigeria-2017*	64.1	5.2	26.3	36.0	32.6	0.0	0.1	99.9	0.2	11.1	88.8
Tajikistan-2014	61.0	7.1	20.2	27.6	45.2	0.0	0.0	100.0	26.0	53.0	21.0
SDI											
Kenya-2012	57.7	2.8	48.5	22.8	25.9	–	–	–	17.3	56.9	25.8
Kenya-2018	57.4	–	–	–	–	1.3	11.7	87.0	12.4	49.9	37.6
Madagascar-2016	63.2	3.4	30.0	27.9	38.7	2.7	11.7	85.6	26.6	54.5	18.9
Mozambique-2014	48.2	9.6	59.3	18.6	12.4	7.7	45.8	46.5	44.9	32.8	22.3
Niger-2015	59.0	8.3	42.6	30.3	18.8	20.4	61.3	18.3	4.8	52.3	42.9
Nigeria-2013	64.1	1.7	29.6	34.3	34.3	3.0	88.0	8.9	3.7	16.3	80.0
Senegal-2010	69.5	4.7	34.1	23.4	37.8	9.6	17.9	42.3	0.0	53.5	46.5
Sierra Leone-2018	77.9	–	–	–	–	1.6	37.8	60.7	0.9	27.2	71.9
Tanzania-2014	67.2	3.5	28.0	29.8	38.7	19.2	56.1	24.7	27.8	29.2	43.0
Tanzania-2016	68.3	5.9	38.4	23.7	31.9	12.9	59.1	28.1	21.1	37.2	41.7
Togo-2013	51.5	3.6	38.0	29.2	29.2	31.4	46.0	22.7	5.0	31.7	63.3
Uganda-2013	61.1	7.5	52.2	23.3	17.0	–	–	–	17.5	50.0	32.5
**Total**	**60.4**	**5.5**	**38.3**	**28.6**	**27.6**	**8.5**	**42.7**	**48.8**	**11.7**	**42.3**	**46.0**

Notes: For cadres, Doctor=doctors or clinical officers, Nurse=nurses or midwives, Other=other health workers. See online supplemental appendix table A7 for detailed information on the type of workers included in each cadre. Workers’ education is not available in Kenya SDI 2012 and Uganda SDI 2013. Workers’ age is not available in Sierra Leone SDI 2018 and Kenya SDI 2018.

* Nigeria 2017 is an RBF endline survey.

LMICs, low- and middle-income countries; PHC, primary healthcare; RBF, results-based financing; SDI, Service Delivery Indicators.

Salary delays were commonly reported but highly variable across countries (figure 1). Averaged across countries, 37% of health workers experienced a delay in the previous month, ranging from 2% in Lesotho (the Lesotho value reflects a 12-month recall item converted to a lower-bound previous-month equivalent (reported prevalence ÷ 12) and likely underestimates true previous-month prevalence given persistence in delays; see Methods and figure 1 note.) to 83% in Uganda. In countries with multiple data rounds, delay rates fluctuated over time, reflecting a mix of real change and measurement differences. For example, in Kenya, the delay rate increased from 22% in 2012 when the survey semi-randomly sampled 15 counties to 46% in 2018 when all 47 counties were covered; in Tanzania, the rate decreased from 60% in 2014 to 20% in 2016 when the 2016 survey included more lower-cadre workers. Because between-round differences may reflect both true variation and changes in recall, survey modules or sampling frames, we treat them descriptively and avoid trend interpretation.

### Reasons and length of salary delay

Table 2 summarises health workers’ self-reported causes and durations of salary delays. The primary reasons reported by public-sector primary care workers were lack of funds and administrative issues. Specifically, 86% of health workers in Tajikistan, 61% in CAR and 44% in Nigeria cited lack of funds as the main cause. Some reasons mentioned included low federal allocation of budget, economic recession and project termination. (These options originally belonged to the ‘unclassified’ category but were recoded into ‘lack of funds’ due to their relevance to budget issues, while not being the only contributing factors in this category.)

**Table 2:** Reasons and length of salary delay among public-sector PHC workers

Reasons for delay	CAR 2012	Cameroon 2012	Gambia 2014	Nigeria* 2017	Tajikistan 2014	Delay >1 month
Lack of funds	61.1%	12.3%	11.1%	43.6%	86.1%	27.9%
Administrative issue	12.8%	43.9%	33.3%	19.5%	6.5%	28.0%
Withheld for debts	0.0%	0.0%	0.0%	2.2%	2.3%	12.2%
Performance/absence	5.4%	0.0%	0.0%	0.3%	0.0%	15.8%
Unclassified	9.3%	3.5%	0.0%	0.6%	0.0%	22.2%
Unexplained	11.3%	40.4%	55.6%	51.0%	22.7%	39.7%
**Total**	257	57	9	1662	216	32.6%

Note: Data on reasons and lengths for salary delay came from five RBF surveys. One SDI survey (Nigeria 2013) also reported salary delay but was not listed here due to different categorization–on average 82% cited the main reason as late release of funds and 18% cited issues with bank processing delays.

* Several responses initially classified as ‘unclassified’ in Nigeria were recoded into other categories based on their content, as follows: 7 cases of ‘don't know’ or ‘no reason’ were recoded as unexplained; 5 cases citing reasons such as low allocation, economic recession, low federal allocation, small monthly allocation or project termination were recoded as ‘lack of funds’; and 25 cases mentioning bank verification number, sanction due to results-based financing, new staff, volunteer status or omission from payment schedule were recoded as ‘administrative issues’.

PHC, primary healthcare; RBF, results-based financing.

Administrative challenges emerged as another significant cause, with 44% of health workers in Cameroon and 33% in Gambia attributing delays to this issue. Detailed reasons included facility sanctions from RBF schemes, omission from payment rolls and special employment statuses such as being a volunteer (Workers of all contract types were retained in the analysis, including those who reported their employment category as ‘volunteer’. Most (95 out of 98) of these self-reported ‘volunteers’ did indicate receiving salaries.) or a new employee. In Nigeria, bank processing delays were frequently reported, with 18% of workers in the SDI 2013 survey citing this issue, while the 2017 RBF survey identified challenges with the bank verification number system.

Less commonly reported reasons included salary withholding for debt repayment or delays tied to performance or absence, indicating these are not predominant concerns. However, significant transparency gaps exist in Gambia and Nigeria, where nearly 40% of workers experiencing salary delays were unaware of the reasons.

On average, 32.6% workers reported delays lasting longer than 1 month. The length of delays was closely tied to the reported reasons. Workers citing systemic issues like lack of funds or administrative issues experienced longer delays, with 28% reporting delays exceeding 1 month. In contrast, delays for reasons like withheld salaries for debts or performance/absence-related issues were shorter, with over 85% reporting delays of less than a month. A significant proportion (40%) of workers reported delays lasting more than a month with no clear explanation.

### Salary delay patterns by individual and facility characteristics

**Figure 2:**
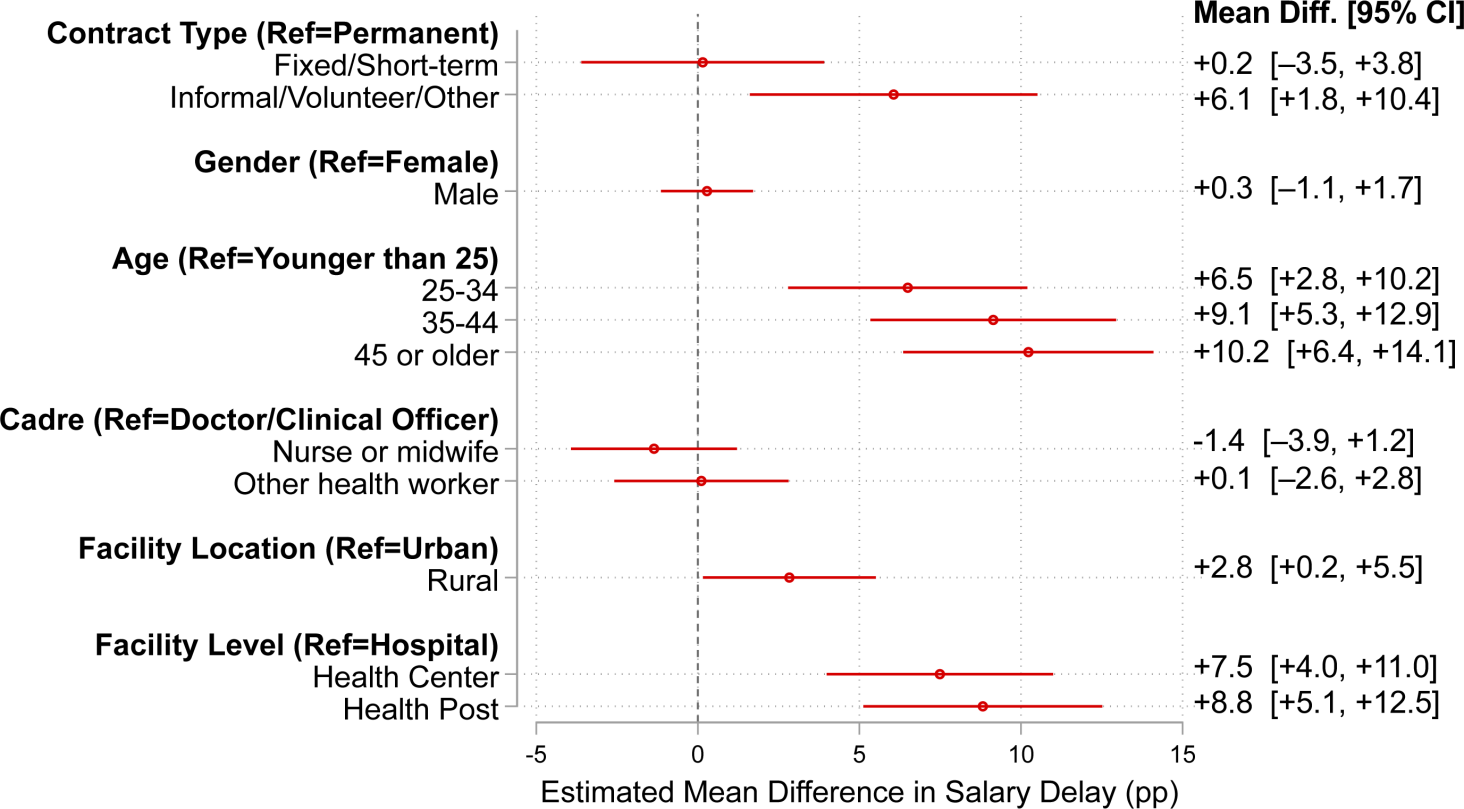
Salary delay patterns by individual and facility characteristics. Note: The analysis includes 22 003 PHC workers from 16 LMICs. Estimated mean differences are from the linear probability models and are interpreted as the adjusted absolute difference (pp) in the probability of reporting a salary delay for each category relative to the indicated reference group, holding all other covariates and fixed effects constant. Models control for worker characteristics (contract type, gender, age, cadre), facility attributes (location and level) and fixed effects for country, year and data source. Standard errors are clustered at the facility level. Missing covariate values were handled by multiple imputations with chained equations. Reference groups are indicated in parentheses. LMICs, low- and middle-income countries; MD, mean difference; PHC, primary healthcare; pp, percentage points.

Figure 2 shows adjusted MDs in the probability of salary delay by worker and facility characteristics (95% CIs). At the individual level, salary delays were more prevalent among health workers without formal contracts. Relative to workers with permanent and pensionable contracts, workers with informal, volunteer or other non-traditional contracts were 6.1 pp more likely to report a delay (95% CI 1.8 to 10.4 pp). Considering health worker age, lower delay rates were concentrated in the small subgroup of workers under age 25; across the three main age bands (25–34, 35–44 and ≥45), delay rates are broadly similar and higher than among workers under 25. No significant differences in salary delay were detected across gender or cadre.

Facility location and level were also associated with salary delay—on average, delays were reported more often in rural facilities compared with urban ones (+2.8 pp, 95% CI +0.2 to +5.5 pp) and at lower-level facilities such as health centres (+7.5 pp, 95% CI +4.0 to +11.0 pp) and health posts (+8.8 pp, 95% CI +5.1 to +12.5 pp) compared with hospitals. Sensitivity analysis using the missing indicator approach yielded similar results (online supplemental appendix figure A2).

### Relationship between salary delay and health worker satisfaction and performance

**Figure 3:**
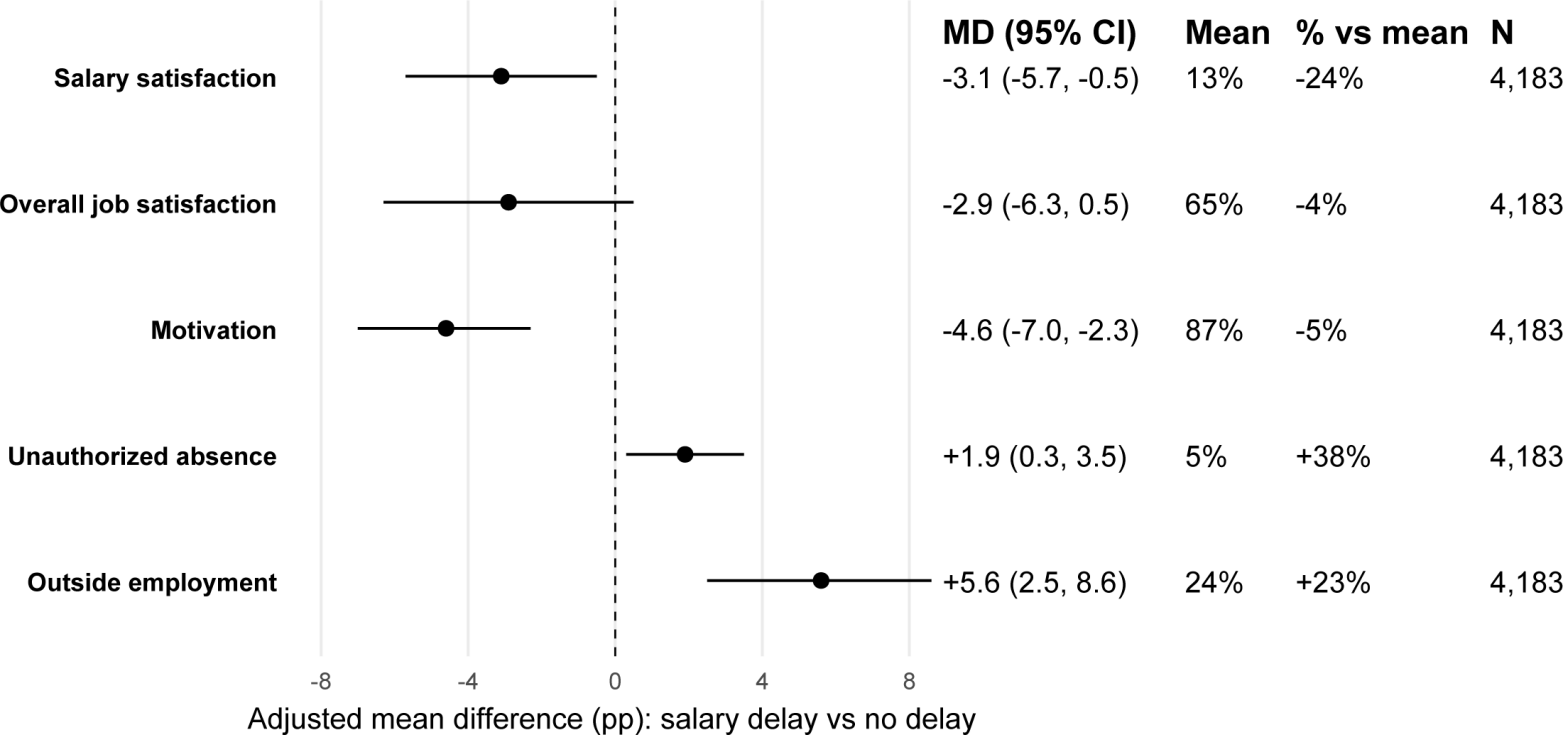
Association between salary delay and health worker behaviour. Note: The analysis includes 4183 PHC workers with complete data on all outcomes from five RBF surveys to ensure consistent sample sizes across outcomes. Estimated MDs are from the linear probability models and are interpreted as the adjusted absolute difference (pp) in the probability of each outcome (satisfied with salary, satisfied with job overall, feeling motivated, unauthorised absence, outside employment) for health workers reporting a salary delay vs those not, holding all other covariates and fixed effects constant. The ‘Mean’ column gives the outcome mean in this analytic sample; ‘% vs mean’ expresses the percent change relative to that mean. Models control for worker characteristics (contract type, gender, age, cadre), facility attributes (location and level), and fixed effects for country, year and data source. Standard errors are clustered at the facility level. Missing covariate values were handled by multiple imputations with chained equations. Results are similar without the balanced-N restriction. MD, mean difference; PHC, primary healthcare; pp, percentage points; RBF, results-based financing.

We further examined the associations between salary delays and health worker behaviours, including satisfaction, motivation, unauthorised absenteeism and outside employment in the RBF subsample. Figure 3 presents the estimated MD, mean value, percent change relative to mean and the sample size for each outcome. On average, 65% of PHC workers reported being satisfied with their job, while only 13% reported being satisfied with their salary. Despite widespread salary dissatisfaction, 87% reported feeling motivated to work. Salary delay was robustly negatively associated with these outcomes. Experiencing a salary delay was associated with a 3.1 pp reduction in the probability of being satisfied with one’s salary (−3.1 pp, 95% CI − 5.7 to −0.5 pp) and a 4.6 pp reduction in the probability of feeling motivated at work (−4.6 pp, 95% CI −7.0 to −2.3 pp), the equivalent of a 24% and 5% decrease in salary satisfaction and motivation, respectively.

On average, 5% of respondents had unauthorised absences during working hours. Salary delays were associated with a 1.9 pp increase in probability of unauthorised absenteeism (+1.9, 95% CI +0.3 to +3.5 pp), a 40% increase relative to the mean. 24% reported having another job to supplement income outside the health facility. Salary delays were associated with a 5.6 pp increase in probability of outside employment (+5.6 pp, 95% CI +2.5 to +8.6 pp), a 24% increase relative to the mean. Results for the leave-one-out analysis (online supplemental appendix table A8) and using the missing indicator approach (online supplemental appendix figure A3) were consistent with the main findings.

## Discussion

Using data from 22 003 health workers across 16 LMICs, this study examined the prevalence and factors associated with salary delays among the public PHC workforce. Our analysis yielded three key findings. First, salary delays were highly prevalent, with an average of 37% workers experiencing delays in receiving their last base salary, with substantial cross-country variation. Workers mainly attributed these delays to funding shortages and administrative issues. Second, salary delays are disproportionately concentrated among workers without formal contracts and in lower-level or rural facilities. Lastly, these delays were associated with reduced salary satisfaction, motivation, increased absenteeism and outside employment. Taken together, these patterns indicate that payment timeliness reflects core PFM constraints (eg, cash management, payroll administration) and can blunt otherwise well-designed performance or quality reforms. Ensuring salary timeliness is therefore a precondition for sustained workforce performance.^22 23^

Frequent salary delays in the public sector reveal a critical deficiency in PHC payment systems. Our findings align with qualitative studies linking inconsistent payments among health workers to dissatisfaction, financial instability and intent to leave one’s role across LMICs.^34^,^37^ Timely, predictable payment is linked to better performance and may show health workers that they are valued.^34^ Improving payment reliability could increase workers’ financial security and improve their confidence in handling future financial shocks. This, in turn, may reduce dissatisfaction, boost motivation and improve performance and retention, all key elements to improving service delivery.

Our findings on the reasons and length of salary delays indicate systemic issues as primary drivers. Beyond budget constraints, respondents frequently cited administrative bottlenecks (eg, payroll errors and bank processing issues), highlighting the need for clearer procedures and digitisation.^38^ These patterns echo evidence linking delays to systemic inefficiencies and leakage in decentralised transfers.^37^ Interestingly, in the only RBF endline survey included in this study, some workers cited RBF sanctions as reasons for base-salary delays, even though RBF mechanisms are designed as bonuses and should not affect base-salary disbursement. This disconnect aligns with previous research showing that poor RBF implementation, which causes delayed or opaque payments, can demotivate staff and foster misunderstandings, contrary to its original aims.^27 39 40^ These cash-to-payroll issues are fixable: when implemented effectively, treasury cash consolidation (eg, a single treasury account) and routine reconciliation of payroll with personnel records can improve payment timeliness, as reflected in PFM guidance and country experience.^41 42^ In parallel, clear communication is crucial to ensure all stakeholders understand RBF’s operational modalities and its separation from base salaries.

Our findings reveal disproportionate salary delays among workers without formal contracts and in rural and lower-level facilities, aligning with evidence on labour exploitation of informal health workers and rural-urban disparities in workforce distribution and working conditions.^43 44^ Contributing factors likely include weaker bargaining power under informal contracts, financial constraints, administrative inefficiencies and geographic isolation.^43 44^ Addressing these systemic inequities requires integrating timely payments into workforce strategies, strengthening payroll and cash-management systems and ensuring equitable resource allocation across all facility types and worker categories.

Our estimates on the association between salary delay and workers’ negative feelings and disruptive behaviours support prior findings from performance-based financing programmes in LMICs, where delays in financial incentives and lack of transparency in the distribution process were reported to lead to feelings of unfairness and distrust among health workers.^3945^,^47^ The relatively large effect sizes on unauthorised absenteeism and outside employment suggest that health systems should consider how payment challenges may contribute to performance issues that affect quality of care and patient outcomes.

Because the bottlenecks we document sit within routine PFM processes, policy responses should pair financing with accountability in the budget-execution chain.^24^ Global financing partners concerned with workforce performance could consider linking elements of sector support to verifiable assurances of timely salary payments (eg, routine tracking and reporting of on-time payment rates), with proportionate oversight and safeguards for fiscal flexibility. To track progress, salary timeliness should also be measured routinely. High-profile facility surveys (eg, Service Provision Assessments) could consider including a brief, standardised module on receipt and timing of base salaries and allowances, with clear recall periods and identifiers for facility level and location. Such data would enable disaggregated tracking and complement administrative sources that are often incomplete or sensitive.

Our analysis has several limitations. First, all key measures are self-reported and subject to social desirability or recall bias; importantly, health workers may not know the true reason for a delay given limited visibility into payroll and treasury processes, so reported reasons should be read as perceptions and interpreted cautiously. Second, comparability can be affected by recall-period differences (Lesotho, 12 months; Burkina Faso, 3 months) and cross-round measurement variation (eg, sampling frames, modules); for these cases, we present recall-standardised values as lower-bound previous-month estimates and interpret cross-round changes cautiously. Third, because facility-based surveys capture only those present on interview days, workers with prolonged absence may be under-represented. If salary delays are linked to higher absence or outside employment, this selection may underestimate the associations between delays and worker outcomes. Fourth, the cross-sectional design identifies associations rather than causality. Finally, the study lacked granular data on payment attributes like frequency, mode (eg, cash, electronic transfer), level, distribution process and administrative responsibility—crucial factors for designing effective payment systems that support worker retention and motivation. Future work that follows salary flows over time and records these attributes would enable stronger inference and deeper insights into workforce payment system challenges.

Overall, salary delays were prevalent among public-sector PHC workers across 16 LMICs during 2010–2018, particularly in rural and lower-level health facilities. These delays were associated with reduced motivation and satisfaction, along with increased instances of unauthorised absenteeism and outside employment. Addressing delays requires diagnosing bottlenecks across administrative tiers and improving cash-to-payroll execution. These findings underscore the need for system-level interventions to reduce salary delays as part of broader health system strengthening. Future work should document payment processes and managerial practices in more detail and incorporate routine measurement of salary timeliness to enable disaggregated tracking and accountability, and examine whether similar patterns occur in private facilities.

## Conclusion

Salary delays are a pervasive challenge for the public-sector PHC workforce in LMICs and are associated with lower salary satisfaction, motivation and performance. Targeted action is needed where delays are most concentrated, specifically among workers without formal contracts and in rural or lower-level facilities, together with system-level improvements in cash-to-payroll execution. Routine measurement and thorough documentation of payment processes will support accountability and help identify actionable strategies to strengthen health systems and advance universal health coverage.

## Supplementary material

10.1136/bmjgh-2024-017742 online supplemental file 1

## Data Availability

Data are available in a public, open access repository.
