# Supplementary material for: Salary delays among public sector primary care workers: evidence from facility surveys across 16 low- and middle-income countries
Source: BMJ Glob Health. 2025 Dec 11;10(12):e017742. doi: 10.1136/bmjgh-2024-017742 (PMC12699552; doi:10.1136/bmjgh-2024-017742)
Supplement: online supplemental file 1 [file bmjgh-10-12-s001.docx]

**Supplemental Materials for “Salary Delays among Public Sector Primary Care Workers: Evidence from Facility Surveys across 16 Low- and Middle-Income Countries****”**

[1 Description of Sample, Exposure, and Outcomes 2](#_Toc209804668)

[Table A1. Geographic, Facility, and Health Worker Coverage of Included Surveys 2](#_Toc209804669)

[Table A2. Measurement Variations and Harmonization Approaches for Key Variables 3](#_Toc209804670)

[Table A3: Governance Structures and Health System Functions for Study Countries 5](#_Toc209804671)

[Table A4. Cadre Classification Process 8](#_Toc209804672)

[Figure A1. Sample Selection Process 10](#_Toc209804673)

[2 Analysis 11](#_Toc209804674)

[2.1 Model Specifications 11](#_Toc209804675)

[2.1.1 Model evaluating the associations between salary delays and worker and facility characteristics 11](#_Toc209804676)

[2.1.2 Model evaluating the associations between health worker behaviors and salary delays 11](#_Toc209804677)

[2.2 Multiple Imputation Methodology 12](#_Toc209804678)

[3 Descriptive Results 13](#_Toc209804679)

[Table A5. Sample Characteristics and Composition for Primary Analytical Sample 13](#_Toc209804680)

[Table A6. Sample Characteristics and Composition for RBF Subsample 14](#_Toc209804681)

[Table A7. Indicator Availability and Percent Missing by Survey Wave 15](#_Toc209804682)

[4 Robustness checks 20](#_Toc209804683)

[Figure A2. Salary Delay Patterns by Individual and Facility Characteristics: Sensitivity Analysis Using Missing Indicator Method for Covariates 20](#_Toc209804684)

[Figure A3. Salary Delay and Health Worker Behaviors Association: Sensitivity Analysis Using Missing Indicator Method for Covariates 21](#_Toc209804685)

[Table A8. Salary Delay and Health Worker Behaviors Association: Leave-One-Out Analysis Results 22](#_Toc209804686)

# **1 Description of Sample, Exposure, and Outcomes**

## **Table A1. Geographic, Facility, and Health Worker Coverage of Included Surveys**

| **Country** | **Year** | **Data Source** | **Geographic Coverage** | **Facility Coverage** | **Health worker Coverage** | **Sampling Notes** |
| --- | --- | --- | --- | --- | --- | --- |
| Burkina Faso | 2013-2014 | RBF | 6 regions (Centre-Nord, Nord, Sud-Ouest, Centre-Est, Boucle du Mouhoun, Centre-Ouest) | All health facilities in targeted districts | All health personnel from regional, district, and local levels | - |
| Central African Republic | 2012 | RBF | 7 prefectures | Public and private not-for-profit hospitals, health centers, and health posts | Random sample of clinical and lay health workers with MCH responsibilities | Regions chosen due to poor outcomes; not nationally representative |
| Cameroon | 2012 | RBF | 14 health districts in North-West, South-West and East regions | All public District Medical Centers, Integrated Health Centers, and District Hospitals | Random sample of clinical health workers with MCH responsibilities | One-day facility visits due to budget constraints; sample limited to providers present on survey day |
| Gambia | 2014 | RBF | Upper River, Central River and North Bank West Regions | All health facilities in the study area | Head of health facility, all health workers in MCH services | Not nationally or regionally representative; covers less developed regions |
| Kenya | 2012, 2018 | SDI | National | All primary-level care facilities | Random sample of clinical health workers | - |
| Lesotho | 2015 | RBF | 4 districts (Mafeteng, Mohale's Hoek, Mokhotlong, Thaba-Tseka) | All health centers managed by the Ministry of Health and Christian Health Association of Lesotho | Up to 3 health workers with MCH responsibilities per facility | 3 health centers randomly excluded, 1 inaccessible. |
| Madagascar | 2016 | SDI | National | All primary-level care facilities | Random sample of clinical health workers | - |
| Mozambique | 2014 | SDI | National | All primary-level care facilities | Random sample of clinical health workers | - |
| Niger | 2015 | SDI | National | All primary-level care facilities | Random sample of clinical health workers | - |
| Nigeria | 2013 | SDI | National | All primary-level care facilities | Random sample of clinical health workers | - |
|  | 2017 | RBF | 6 states (Adamawa, Benue, Nasarawa, Ogun, Ondo, Taraba) | Primary and secondary health facilities | Up to 3 health workers per facility | - |
| Senegal | 2010 | SDI | National | All primary-level care facilities | Random sample of clinical health workers | - |
| Sierra Leone | 2018 | SDI | National | All primary-level care facilities | Random sample of clinical health workers | - |
| Tajikistan | 2014 | RBF | 5 districts in Sughd region, 11 districts in Khatlon region | Rural Health Centers (RHC) and health houses (HH) | Random sample of 4 health workers per RHC and HH | In Khatlon, 4 facilities replaced due to absence of RHC and referring HH |
| Tanzania | 2014, 2016 | SDI | National | All primary-level care facilities | Random sample of clinical health workers | - |
| Togo | 2013 | SDI | National | All primary-level care facilities | Random sample of clinical health workers | - |
| Uganda | 2013 | SDI | National | All primary-level care facilities | Random sample of clinical health workers | - |

Notes: Coverage and sampling details extracted from survey reports. RBF = Results-Based Financing Survey. SDI = Service Delivery Indicator Survey. MCH = Maternal and Child Health.

## **Table A2. Measurement Variations and Harmonization Approaches for Key Variables**

| **Variable  Group** | **Measurement in Survey** | | | | **Harmonized Measurement** |
| --- | --- | --- | --- | --- | --- |
|  | **Survey** | **Questions** | **Options** | **Modification** |  |
| Salary Delay | All SDI except for Senegal | Did you experience a delay in receiving your last salary? | Yes/No | - | Yes = 1; No = 0 |
|  | Senegal; Cameroon; CAR; Gambia; Tajikistan;  Nigeria | Last month, how many days late was you salary delayed? | Received on time = 0;  Number of days;  Still not received = 77 | Yes = any number larger than 0 or still not received  No = 0 |  |
|  | Lesotho | In the last 12 months, did you receive all your due salary according to the payment schedule? | Yes/No | - |  |
|  | Burkina Faso | In the last 3 months, have you received your salary in totality? | Yes, without delays = 1; Yes, but with delays = 2; No = 3 | Yes = 2, 3; No = 1 |  |
| Salary Satisfaction | Camerooon; Lesotho; CAR; Tajikistan; Nigeria | How satisfied are you with your salary? | Satisfied = 1; Indifferent = 2; Unsatisfied = 3; N/A = 4, 9 | Satisfied = 1; Unsatisfied = 2, 3 | Satisfied = 1; Unsatisfied =0 |
| Overall Job Satisfaction | Cameroon; Lesotho; CAR; Tajikistan; Nigeria | Overall, how satisfied are you with your job? | Satisfied = 1; Indifferent = 2; Unsatisfied = 3 | Satisfied = 1; Unsatisfied = 2, 3 | Satisfied = 1; Unsatisfied =0 |
| Motivation | Cameroon | These days, I feel motivated to work as hard as I can. | Most of the time = 1; More than half of the time = 2; Less than half of the time = 3; Only rarely = 4; Never = 5; N/A = 9 | Motivated = 1, 2; Unmotivated = 3, 4, 5; | Motivated = 1; Unmotivated = 0 |
|  | CAR | These days, I feel motivated to work as hard as I can. | Most of the time = 1; More than half of the time = 2; Less than half of the time = 3; Never = 4 | Motivated = 1, 2; Unmotivated = 3, 4 |  |
|  | Lesotho | These days, I feel motivated to work as hard as I can. | Strongly Disagree = 1; Disagree = 2; Agree = 3; Strongly Agree = 3 | Motivated = 3, 4; Unmotivated = 1, 2 |  |
|  | Tajikistan | This health facility inspires me to do my very best on the job. | Most of the time = 1; More than half of the time = 2; Less than half of the time = 3; Only rarely = 4; Never = 5; | Motivated = 1, 2; Unmotivated = 3, 4, 5 |  |
|  | Nigeria | Overall, I am motivated to do my job. | Strongly Disagree = 1; Disagree = 2; Neither Agree nor Disagree = 3; Agree = 4; Strongly Agree = 4 | Motivated = 4, 5; Unmotivated = 1, 2, 3 |  |
| Unauthorized absenteeism | Cameroon; Lesotho; CAR; Tajikistan; Nigeria | Have you ever been absent from work without authorized leave? | Yes/No | - | Yes = 1; No = 0 |
| Outside employment | Cameroon; Lesotho; CAR; Tajikistan; Nigeria | Do you have any other job or activity to supplement your income from this health facility? | Yes/No | - | Yes = 1; No = 0 |

Notes: SDI = Service Delivery Indicator Survey. CAR = Central African Republic

## **Table A3: Governance Structures and Health System Functions for Study Countries**

| **Country** | **Governance Structure** | **Subnational Divisions** | **Subnational Division Responsibilities for Health** | | |
| --- | --- | --- | --- | --- | --- |
|  |  |  | **Level 1** | **Level 2** | **Level 3** |
| Burkina Faso | Unitary presidential system | Level 1: 13 regions Level 2: 45 provinces Level 3: 351 communes | Participating in the building and management of basic and intermediate health facilities; valuing traditional pharmacopoeia | N/A | Primary health care (health centers), preventive health, cemeteries, funeral services |
| Central African Republic | Decentralized unitary state | Level 1: 20 prefectures and one autonomous commune Level 2: 84 sub-prefectures | N/A | N/A | N/A |
| Cameroon | Semi-presidential unitary republic | Level 1: 10 regions Level 2: 14 urban communties Level 3: 360 communes | Maintenance and operation of regional health centers; preventive health plans; provision of health services in accordance with national policy | Monitoring and control of industrial waste | Maintenance and operation of sanitary facilities; Hygiene control of local industries and health services (creation and management of District Medical Centres) |
| Gambia | Multi-party presidential system | Level 1: 2 municipalities and 6 local government areas  Level 2: 43 districts | Primary healthcare (medical centres); preventative healthcare; public health services | N/A | N/A |
| Kenya | Unitary state | Level 1: 8 provinces Level 2: 47 counties Level 3: 4 cities and 55 municipalities | N/A | County health facilities and pharmacies; ambulance services; promotion of primary healthcare; licensing and control of food safety; cemeteries, funeral parlours and crematoria (shared) | Cemeteries and crematoria, ambulance services, health facilities |
| Lesotho | Constitutional monarchy, parliamentary democracy | Level 1: 10 districts Level 2: 128 community council | N/A | N/A | N/A |
| Madagascar | Semi-presidential unitary state | Level 1: 23 regions Level 2: 119 districts Level 3: 1695 communes | Managing regional public health facilities (Regional Hospital Centres) | N/A | Constructing and managing basic public health facilities and infrastructure (shared) |
| Mozambique | Democratic republic | Level 1: 10 provinces and 1 capital city Level 2: 129 districts Level 3: 394 administrative posts | Primary care and maintenance of hospitals (shared with national government) | N/A | N/A |
| Niger | Unitary semi-presidential state | Level 1: 8 regions Level 2: 63 departments Level 3: 266 municipalities | Constructing, maintaining and managing regional hospitals, maternal and child centres and other specialised regional centres | N/A | Social assistance to vulnerable groups; funeral services; primary healthcare centers |
| Nigeria | Federal republic | Level 1: 36 states and 1 Federal Capital Territory Level 2: 768 local governments authorities | General hospitals | Primary healthcare (medical centers), preventive healthcare (shared) | N/A |
| Sierra Leone | Unitary constitutional republic | Level 1: 4 provinces and 1 area Level 2: 16 districts Level 3: 190 chiefdoms | N/A | Primary and secondary health (health service delivery, health promotion, drug control and prevention); hospital management (shared with national government); health protection | N/A |
| Senegal | Unitary republic | Level 1: 14 regions  Level 2: 43 departments  Level 3: 557 communes (of which 5 cities) | N/A | Hospitals | Primary healthcare (health centers), preventive health |
| Tajikistan | Unitary presidential republic | Level 1: 4 regions and 1 capital city Level 2: 47 districts and 18 cities Level 3: 368 commues or municipalities | Hospitals; Primary healthcare (medical centres); Preventive healthcare | N/A | N/A |
| Tanzania | Democratic unitary republic (Mainland and Zanzibar) | Level 1: 31 regions Level 2: 169 districts Level 3: divisions  Level 4: wards | N/A | N/A | Primary care; Hospitals, Health protection |
| Togo | Unitary state | Level 1: 5 regions Level 2: 39 prefectures Level 3: 117 communes | Supporting the management of regional hospitals; regional disease prevention policy (shared) | N/A | Hygiene and sanitation measures; dispensaries, basic health units and pharmaceutical depots (shared) |
| Uganda | Democratic unitary republic | Level 1: 4 regions Level 2: 121 districts | N/A | Primary care, hospitals, health protection (discretionary) | N/A |

Note: The information on governance structure, subnational division, and subnational responsibilities for health is derived from the World Observatory on Subnational Government Finance and Investment (SNG-WOFI) fiscal database, incorporating the most up-to-date available data. To ensure consistency with the survey dataset, the levels of subnational divisions and their corresponding responsibilities for health have been adjusted by the authors. The legislation in some countries (e.g., Tajikistan) lacks a clear definition of functions and responsibilities for the various levels of subnational governments, posing a challenge in precisely delineating these roles in the analysis. N/A = Not available.

## **Table A4. Cadre Classification Process**

To harmonize health worker data across RBF and SDI country samples, we classified public sector facility-based primary care health workers into four broad cadres: “Doctors/Clinical Officers”, “Nurses”, “Health workers”, and “Other”. The "Other" category, which includes workers who do not engage directly with patients (e.g., technicians, administrative staff, accountants), was dropped from our analysis. Note that the "Health Worker" category in SDI country samples might include some non-health-related facility workers due to difficulties in differentiating them from health workers. The table below details the types of workers included under each cadre in our analysis:

| **Country** | **Year** | **Source** | **Doctor/Clinical Officer** | **Nurse** | **Health Worker** |
| --- | --- | --- | --- | --- | --- |
| Kenya | 2012 | SDI | 1. Doctors 2. Clinical Officers | 1. Nurses 2. Midwives 3. BSc. Nurses | 1. Nurse Aides 2. Para-professionals 3. Pharmacists |
|  | 2018 |  | 1. Doctors (specialist and general medical doctors) 2. Clinical Officers | Nurses | Other |
| Mozambique | 2014 | SDI | 1. Director 2. Doctor (Specialist) 3. Doctor (Generalist) 4. Clinical Officer 5. Assistant Clinical Officer | 1. Nurse 2. Midwife | 1. Laboratory technician 2. Health worker 3. Other |
| Niger | 2015 | SDI | Doctor | 1. Nurse 2. Midwife 3. Delivery Nurse 4. Certified Nurse 5. Deputy Nurse 6. Medical Assistant, Nursing | 1. Medical Assistant, surgery 2. Medical Assistant, anesthesiology 3. Laboratory technician 4. Hygiene technician 5. Social Assistant 6. Basic health workers 7. Hygiene workers 8. Deputy social assistant 9. Community Health Worker 10. Other |
| Nigeria | 2013 | SDI | Doctor | Nurses and Midwives | 1. Community Health Workers 2. Paraprofessionals |
|  | 2017 | RBF | 1. Doctor 2. Medical Officer | Nurses and Midwives | 1. Health assistants >1 year 2. Other non-clinical 3. Public Health Nurse 4. Community Health Officer (CHO) 5. Community Health Extension Worker (CHEW) 6. Jr. CHEW |
| Sierra Leone | 2018 | SDI | Doctor | Nurses and Midwives | Community Health Officer (CHO)/Community Health Assistant (CHA) |
| Senegal | 2010 | SDI | Doctor | Nurses and Midwives | 1. Community Health Workers 2. Paraprofessionals |
| Tanzania | 2014 | SDI | 1. Medical Doctor (Specialist) 2. Doctor (Generalist) 3. Medical Officer 4. Asst Medical Officer | Nurse/Nurse Midwife | 1. Lab/Pharmacy 2. Public Health Officer 3. Medical Att./Nurse Assistant 4. Maternal and Child Health Aide 5. Rural Medical Aides 6. Other |
| Togo | 2013 | SDI | 1. Specialist, Doctor 2. Medical Officer (diploma) | 1. Nurse (diploma) 2. Nurse auxiliary (diploma) 3. Nurse (no diploma) 4. Midwives | 1. Medical officer (no diploma) 2. Trained birth attendant (diploma) 3. Trained birth attendant (no diploma) 4. Nurse’s aide 5. Biological engineer 6. Laboratory technician 7. Hygiene worker 8. Other |
| Tajikistan | 2014 | RBF | 1. Family Physician 2. Obstetrician/Gynecologist 3. Clinical intern/Jr. doctor 4. Cardiologist 5. ENT Doctor 6. Pediatrician 7. Surgeon 8. Infectiologist | 1. Midwife 2. Family Nurse | 1. Dentist 2. Physiotherapist 3. Therapeutist |
| Burkina Faso | 2013 | RBF | 1. General Practitioner 2. Specialist 3. Anesthesia health officer 4. Gyneco-obstetric health officer 5. Surgical health officer 6. Pediatric health officer 7. ENT health officer | 1. Nursing and midwifery Associate 2. State Registered Nurse 3. Registered Nurse 4. State Midwife 5. Auxiliary Midwife 6. Certified Midwife | 1. Mobile Health Officer |
| Central Africa Republic | 2012 | RBF | 1. Doctor 2. Major – Pediatrics 3. Major – Maternity 4. Major – Internal Medicine 5. Major – Operating room 6. Head of external consulations 7. Head of health training | 1. Senior Nursing Technician 2. State Registered Nurse (IDE) 3. State-ceritified midwifery nurse 4. IDE Childcare worker | 1. Superintendent 2. Maternal and Child Health/FP Officer 3. Head of ENP Unit 4. Senior Technician in Anesthesia and Resuscitation 5. Other |
| Cameroon | 2012 | RBF | 1. Director/Head of health training 2. Superintendent 3. Major – Pediatrics 4. Major – Maternity 5. Major – Medicine 6. Major – Surgery 7. Other Manager | 1. Head or Principal nurse | 1. Care Coordinator 2. Other |
| Lesotho | 2015 | RBF | Lesotho only included nurses | 1. Nurse clinician 2. Nurse midwife 3. General Nurse 4. Nurse Assistant 5. Other | Lesotho only included nurses |

## **Figure A1. Sample Selection Process**

Only included five RBF surveys that had complete information on satisfaction, motivation, absenteeism, and outside employment.

Dropped 67 non-health workers

Dropped 218 workers with missing/unclear information on levels of health facility

Full sample for descriptive and factorial analysis

(N = 22,003)

RBF subsample for association analysis

(N = 4,183)

Population with complete information on salary delay

(N = 22,288)

# **2 Analysis**

## **2.1 Model Specifications**

### *2.1.1 Model evaluating the associations between salary delays and worker and facility characteristics*

We assessed the associations between salary delays and a set of health worker characteristics as well as facility attributes using a fixed-effects linear probability regression model. The model is specified as below:

$${SalaryDelay}_{ijt}=\alpha_{j}+\beta\cdot\mathbf{X}_{\mathbf{ijt}}+\mu\cdot\mathbf{Z}_{\mathbf{jt}}+\gamma_{t}+\eta_{k}+ \epsilon_{ijt}$$

where ${SalaryDelay}_{ijt}$is a binary indicator representing whether health worker *i* from country *j* in year *t* experienced any delay in receiving their last month’s base salary payment beyond the scheduled pay date. $\mathbf{X}_{\mathbf{ijt}}$ is a vector of individual covariates, including worker cadre (doctor/clinical officer, nurse/midwife, other health workers), employment contract type (permanent, fixed-/short-term, informal/volunteer/other), gender, and age. $\mathbf{Z}_{\mathbf{jt}}$ is a vector of facility-level covariates, including facility location (rural/urban) and level (hospital, health center, health post). $\alpha_{j}$ is a full set of country-specific fixed-effects that control for time-invariant country characteristics, $\gamma_{t}$ is a set of survey-year specific fixed-effects that control for secular trends, and $\eta_{k}$ includes data source fixed effects to control for sampling differences. Standard errors were adjusted for clustering at the facility level.

### *2.1.2 Model evaluating the associations between health worker behaviors and salary delays*

We assessed how salary delay was associated with a range of health worker behavioral indicators, including worker’s satisfaction, motivation, unauthorized absence, and outside employment. We fit the following fixed-effects linear probability regression model specified below:

$$Y_{ijt}=\alpha_{j}+\beta\cdot{SalaryDelay}_{ijt}+\lambda\cdot\mathbf{X}_{\mathbf{ijt}}+\mu\cdot\mathbf{Z}_{\mathbf{jt}}+\gamma_{t}+\eta_{k}+ \epsilon_{ijt}$$

where $Y_{ijt}$represents the outcome variable for health worekr *i* from country *j* in year *t*. ${SalaryDelay}_{ijt}$ is a binary indicator set to 1 if the worker *i* experienced any delay in receiving their last month’s base salary payment beyond the scheduled pay date. $\mathbf{X}_{\mathbf{ijt}}$ is a vector of individual covariates, including cadre (doctor/clinical officer, nurse/midwife, other health workers), employment contract type (permanent, fixed-/short-term, informal/volunteer/other), gender, and age. $\mathbf{Z}_{\mathbf{jt}}$ is a vector of facility-level covariates, including facility location (rural/urban) and level (hospital, health center, health post). $\alpha_{j}$ is a full set of country-specific fixed-effects that control for time-invariant country characteristics, $\gamma_{t}$ is a set of survey-year specific fixed-effects that control for secular trends, and $\eta_{k}$ includes data source fixed effects to control for sampling differences. Standard errors were adjusted for clustering at the facility level. The outcome variables for this model include:

1. Salary satisfaction: A binary variable set to 1 if the health worker reported being satisfied with their salary more than half the time (or a rating above the scale midpoint) and 0 otherwise;
2. Overall Job Satisfaction: A binary variable set to 1 if the health worker reported being satisfied with their job and 0 otherwise.
3. Motivation: A binary variable set to 1 if the worker reported high motivation and 0 otherwise.
4. Unauthorized Absence: A binary variable set to 1 if the worker reported any unauthorized absence from work;
5. Outside Employment: A binary variable set to 1 if the worker reported having any other job or income-generating activity to supplement their primary facility role and 0 otherwise.

## **2.2 Multiple Imputation Methodology**

We used multiple imputation to address missing data on the key independent variables in our analyses. Specifically, we imputed missing values for health worker's contract type, gender, facility location (rural/urban), and age group using the **`mi impute chained`** command in Stata.

For binary variables (worker’s gender and facility location), logistic regression models were used for imputation. For ordinal variables (contract type and age group), we used ordered logistic regression models. The imputation models included worker cadre, facility level, country, year, and data source as predictors to capture the systematic patterns of missingness. Ten imputed datasets were generated, and results were combined using Rubin's rules with the **`mi estimate`** command, allowing us to appropriately account for the uncertainty introduced by missing data during statistical inferences.

To ensure our results were not affected by the multiple imputation approach, we conducted a sensitivity analysis using the missing indicator method. This approach involved creating binary indicator variables for any missing values in the key independent variables and including these indicators in the regression models. The results obtained from this method were consistent with those from the multiple imputation analysis as shown in Figures A2 and A3..

The imputed datasets served as the primary analytical sample to explore the associations between salary delays and worker characteristics, as well as worker behaviors.

# **3 Descriptive Results**

## **Table A5. Sample Characteristics and Composition for Primary Analytical Sample**

| **Country** | **Year** | **Data source** | **Number of health facilities** | **% Hospitals** | **% Health centers** | **% Health posts** | **Number of health workers** |
| --- | --- | --- | --- | --- | --- | --- | --- |
| Burkina Faso | 2013 | RBF | 550 | 6% | 93% | 1% | 1442 |
| CAR | 2012 | RBF | 494 | 7% | 46% | 47% | 701 |
| Cameroon | 2012 | RBF | 153 | 8% | 12% | 80% | 313 |
| Gambia | 2014 | RBF | 24 | 8% | 13% | 79% | 94 |
| Kenya | 2012 | SDI | 155 | 19% | 60% | 21% | 508 |
|  | 2018 | SDI | 1445 | 9% | 23% | 68% | 3473 |
| Lesotho | 2015 | RBF | 31 | 0% | 100% | 0% | 58 |
| Madagascar | 2016 | SDI | 254 | 6% | 68% | 26% | 639 |
| Mozambique | 2014 | SDI | 191 | 20% | 5% | 75% | 741 |
| Niger | 2015 | SDI | 190 | 8% | 31% | 61% | 373 |
| Nigeria | 2013 | SDI | 2013 | 15% | 63% | 21% | 5476 |
|  | 2017 | RBF | 962 | 0% | 100% | 0% | 2373 |
| Senegal | 2010 | SDI | 138 | 0% | 80% | 20% | 619 |
| Sierra Leone | 2018 | SDI | 473 | 4% | 19% | 77% | 1050 |
| Tajikistan | 2014 | RBF | 367 | 0% | 59% | 41% | 1085 |
| Tanzania | 2014 | SDI | 262 | 5% | 21% | 74% | 1045 |
|  | 2016 | SDI | 266 | 4% | 23% | 74% | 1158 |
| Togo | 2013 | SDI | 134 | 12% | 18% | 70% | 439 |
| Uganda | 2013 | SDI | 199 | 1% | 34% | 65% | 416 |
| Total |  |  | 8301 |  |  |  | 22003 |

## **Table A6. Sample Characteristics and Composition for RBF Subsample**

| **Country** | **Year** | **Data source** | **Number of health facilities** | **% Hospitals** | **% Health centers** | **% Health posts** | **Number of health workers** |
| --- | --- | --- | --- | --- | --- | --- | --- |
| CAR | 2012 | RBF | 494 | 7% | 46% | 47% | 701 |
| Cameroon | 2012 | RBF | 145 | 8% | 12% | 80% | 276 |
| Lesotho | 2015 | RBF | 31 | 0% | 100% | 0% | 55 |
| Nigeria | 2017 | RBF | 961 | 0% | 100% | 0% | 2369 |
| Tajikistan | 2014 | RBF | 216 | 0% | 99% | 1% | 782 |
| Total |  |  | 1847 |  |  |  | 4183 |

## **Table A7. Indicator Availability and Percent Missing by Survey Wave**

| **Country** | **Survey year** | **Data source** | **Variable** | **Total sample size (N)** | **Collected?** | **Percent missing (%)** |
| --- | --- | --- | --- | --- | --- | --- |
| Burkina Faso | 2013 | RBF | Salary delay | 1442 | Yes | 0 |
|  | 2013 | RBF | Sex | 1442 | Yes | 0 |
|  | 2013 | RBF | Rural | 1442 | Yes | 1 |
|  | 2013 | RBF | Contract type | 1442 | Yes | 0 |
|  | 2013 | RBF | Age | 1442 | Yes | 0 |
|  | 2013 | RBF | Cadre | 1442 | Yes | 0 |
|  | 2013 | RBF | Facility level | 1442 | Yes | 0 |
|  | 2013 | RBF | Salary satisfaction | 1442 | Yes | 3 |
|  | 2013 | RBF | Overall satisfaction | 1442 | No | - |
|  | 2013 | RBF | Motivation | 1442 | Yes | 39 |
|  | 2013 | RBF | Unaurized absence | 1442 | Yes | 2 |
|  | 2013 | RBF | Outside employment | 1442 | Yes | 0 |
| Central Africa Republic | 2012 | RBF | Salary delay | 701 | Yes | 0 |
|  | 2012 | RBF | Sex | 701 | Yes | 0 |
|  | 2012 | RBF | Rural | 701 | No | - |
|  | 2012 | RBF | Contract type | 701 | Yes | 0 |
|  | 2012 | RBF | Age | 701 | Yes | 5 |
|  | 2012 | RBF | Cadre | 701 | Yes | 0 |
|  | 2012 | RBF | Facility level | 701 | Yes | 0 |
|  | 2012 | RBF | Salary satisfaction | 701 | Yes | 0 |
|  | 2012 | RBF | Overall satisfaction | 701 | Yes | 0 |
|  | 2012 | RBF | Motivation | 701 | Yes | 0 |
|  | 2012 | RBF | Unaurized absence | 701 | Yes | 0 |
|  | 2012 | RBF | Outside employment | 701 | Yes | 0 |
| Cameroon | 2012 | RBF | Salary delay | 313 | Yes | 0 |
|  | 2012 | RBF | Sex | 313 | Yes | 0 |
|  | 2012 | RBF | Rural | 313 | No | - |
|  | 2012 | RBF | Contract type | 313 | Yes | 0 |
|  | 2012 | RBF | Age | 313 | Yes | 2 |
|  | 2012 | RBF | Cadre | 313 | Yes | 0 |
|  | 2012 | RBF | Facility level | 313 | Yes | 0 |
|  | 2012 | RBF | Salary satisfaction | 313 | Yes | 12 |
|  | 2012 | RBF | Overall satisfaction | 313 | Yes | 0 |
|  | 2012 | RBF | Motivation | 313 | Yes | 0 |
|  | 2012 | RBF | Unaurized absence | 313 | Yes | 0 |
|  | 2012 | RBF | Outside employment | 313 | Yes | 0 |
| Gambia | 2014 | RBF | Salary delay | 94 | Yes | 0 |
|  | 2014 | RBF | Sex | 94 | Yes | 0 |
|  | 2014 | RBF | Rural | 94 | No | - |
|  | 2014 | RBF | Contract type | 94 | Yes | 0 |
|  | 2014 | RBF | Age | 94 | Yes | 0 |
|  | 2014 | RBF | Cadre | 94 | Yes | 0 |
|  | 2014 | RBF | Facility level | 94 | Yes | 0 |
|  | 2014 | RBF | Salary satisfaction | 94 | Yes | 0 |
|  | 2014 | RBF | Overall satisfaction | 94 | Yes | 0 |
|  | 2014 | RBF | Motivation | 94 | Yes | 0 |
|  | 2014 | RBF | Unaurized absence | 94 | Yes | 0 |
|  | 2014 | RBF | Outside employment | 94 | No | - |
| Kenya | 2012 | SDI | Salary delay | 508 | Yes | 0 |
|  | 2012 | SDI | Sex | 508 | Yes | 0 |
|  | 2012 | SDI | Rural | 508 | Yes | 0 |
|  | 2012 | SDI | Contract type | 508 | No | - |
|  | 2012 | SDI | Age | 508 | Yes | 3 |
|  | 2012 | SDI | Cadre | 508 | Yes | 0 |
|  | 2012 | SDI | Facility level | 508 | Yes | 0 |
|  | 2012 | SDI | Salary satisfaction | 508 | No | - |
|  | 2012 | SDI | Overall satisfaction | 508 | No | - |
|  | 2012 | SDI | Motivation | 508 | No | - |
|  | 2012 | SDI | Unaurized absence | 508 | Yes | 1 |
|  | 2012 | SDI | Outside employment | 508 | No | - |
|  | 2018 | SDI | Salary delay | 3473 | Yes | 0 |
|  | 2018 | SDI | Sex | 3473 | Yes | 0 |
|  | 2018 | SDI | Rural | 3473 | Yes | 0 |
|  | 2018 | SDI | Contract type | 3473 | No | - |
|  | 2018 | SDI | Age | 3473 | No | - |
|  | 2018 | SDI | Cadre | 3473 | Yes | 0 |
|  | 2018 | SDI | Facility level | 3473 | Yes | 0 |
|  | 2018 | SDI | Salary satisfaction | 3473 | No | - |
|  | 2018 | SDI | Overall satisfaction | 3473 | No | - |
|  | 2018 | SDI | Motivation | 3473 | No | - |
|  | 2018 | SDI | Unaurized absence | 3473 | Yes | 0 |
|  | 2018 | SDI | Outside employment | 3473 | No | - |
| Lesotho | 2015 | RBF | Salary delay | 58 | Yes | 0 |
|  | 2015 | RBF | Sex | 58 | Yes | 0 |
|  | 2015 | RBF | Rural | 58 | No | - |
|  | 2015 | RBF | Contract type | 58 | Yes | 0 |
|  | 2015 | RBF | Age | 58 | Yes | 9 |
|  | 2015 | RBF | Cadre | 58 | Yes | 0 |
|  | 2015 | RBF | Facility level | 58 | Yes | 0 |
|  | 2015 | RBF | Salary satisfaction | 58 | Yes | 3 |
|  | 2015 | RBF | Overall satisfaction | 58 | Yes | 3 |
|  | 2015 | RBF | Motivation | 58 | Yes | 2 |
|  | 2015 | RBF | Unaurized absence | 58 | Yes | 0 |
|  | 2015 | RBF | Outside employment | 58 | Yes | 0 |
| Madagascar | 2016 | SDI | Salary delay | 639 | Yes | 0 |
|  | 2016 | SDI | Sex | 639 | Yes | 0 |
|  | 2016 | SDI | Rural | 639 | Yes | 0 |
|  | 2016 | SDI | Contract type | 639 | No | - |
|  | 2016 | SDI | Age | 639 | Yes | 0 |
|  | 2016 | SDI | Cadre | 639 | Yes | 0 |
|  | 2016 | SDI | Facility level | 639 | Yes | 0 |
|  | 2016 | SDI | Salary satisfaction | 639 | No | - |
|  | 2016 | SDI | Overall satisfaction | 639 | No | - |
|  | 2016 | SDI | Motivation | 639 | No | - |
|  | 2016 | SDI | Unaurized absence | 639 | Yes | 0 |
|  | 2016 | SDI | Outside employment | 639 | No | - |
| Mozambique | 2014 | SDI | Salary delay | 741 | Yes | 0 |
|  | 2014 | SDI | Sex | 741 | Yes | 0 |
|  | 2014 | SDI | Rural | 741 | Yes | 0 |
|  | 2014 | SDI | Contract type | 741 | No | - |
|  | 2014 | SDI | Age | 741 | Yes | 0 |
|  | 2014 | SDI | Cadre | 741 | Yes | 0 |
|  | 2014 | SDI | Facility level | 741 | Yes | 0 |
|  | 2014 | SDI | Salary satisfaction | 741 | No | - |
|  | 2014 | SDI | Overall satisfaction | 741 | No | - |
|  | 2014 | SDI | Motivation | 741 | No | - |
|  | 2014 | SDI | Unaurized absence | 741 | Yes | 0 |
|  | 2014 | SDI | Outside employment | 741 | No | - |
| Niger | 2015 | SDI | Salary delay | 373 | Yes | 0 |
|  | 2015 | SDI | Sex | 373 | Yes | 0 |
|  | 2015 | SDI | Rural | 373 | Yes | 0 |
|  | 2015 | SDI | Contract type | 373 | No | - |
|  | 2015 | SDI | Age | 373 | Yes | 0 |
|  | 2015 | SDI | Cadre | 373 | Yes | 0 |
|  | 2015 | SDI | Facility level | 373 | Yes | 0 |
|  | 2015 | SDI | Salary satisfaction | 373 | No | - |
|  | 2015 | SDI | Overall satisfaction | 373 | No | - |
|  | 2015 | SDI | Motivation | 373 | No | - |
|  | 2015 | SDI | Unaurized absence | 373 | Yes | 0 |
|  | 2015 | SDI | Outside employment | 373 | No | - |
| Nigeria | 2013 | SDI | Salary delay | 5476 | Yes | 0 |
|  | 2013 | SDI | Sex | 5476 | Yes | 0 |
|  | 2013 | SDI | Rural | 5476 | Yes | 0 |
|  | 2013 | SDI | Contract type | 5476 | No | - |
|  | 2013 | SDI | Age | 5476 | Yes | 0 |
|  | 2013 | SDI | Cadre | 5476 | Yes | 0 |
|  | 2013 | SDI | Facility level | 5476 | Yes | 0 |
|  | 2013 | SDI | Salary satisfaction | 5476 | No | - |
|  | 2013 | SDI | Overall satisfaction | 5476 | No | - |
|  | 2013 | SDI | Motivation | 5476 | No | - |
|  | 2013 | SDI | Unaurized absence | 5476 | Yes | 0 |
|  | 2013 | SDI | Outside employment | 5476 | No | - |
|  | 2017 | RBF | Salary delay | 2373 | Yes | 0 |
|  | 2017 | RBF | Sex | 2373 | Yes | 0 |
|  | 2017 | RBF | Rural | 2373 | No | - |
|  | 2017 | RBF | Contract type | 2373 | No | - |
|  | 2017 | RBF | Age | 2373 | Yes | 0 |
|  | 2017 | RBF | Cadre | 2373 | Yes | 0 |
|  | 2017 | RBF | Facility level | 2373 | Yes | 0 |
|  | 2017 | RBF | Salary satisfaction | 2373 | Yes | 0 |
|  | 2017 | RBF | Overall satisfaction | 2373 | Yes | 0 |
|  | 2017 | RBF | Motivation | 2373 | Yes | 0 |
|  | 2017 | RBF | Unaurized absence | 2373 | Yes | 0 |
|  | 2017 | RBF | Outside employment | 2373 | Yes | 0 |
| Senegal | 2010 | SDI | Salary delay | 619 | Yes | 0 |
|  | 2010 | SDI | Sex | 619 | Yes | 0 |
|  | 2010 | SDI | Rural | 619 | Yes | 0 |
|  | 2010 | SDI | Contract type | 619 | No | - |
|  | 2010 | SDI | Age | 619 | Yes | 0 |
|  | 2010 | SDI | Cadre | 619 | Yes | 0 |
|  | 2010 | SDI | Facility level | 619 | Yes | 0 |
|  | 2010 | SDI | Salary satisfaction | 619 | No | - |
|  | 2010 | SDI | Overall satisfaction | 619 | No | - |
|  | 2010 | SDI | Motivation | 619 | No | - |
|  | 2010 | SDI | Unaurized absence | 619 | Yes | 0 |
|  | 2010 | SDI | Outside employment | 619 | No | - |
| Sierra Leone | 2018 | SDI | Salary delay | 1050 | Yes | 0 |
|  | 2018 | SDI | Sex | 1050 | Yes | 0 |
|  | 2018 | SDI | Rural | 1050 | Yes | 0 |
|  | 2018 | SDI | Contract type | 1050 | No | - |
|  | 2018 | SDI | Age | 1050 | No | - |
|  | 2018 | SDI | Cadre | 1050 | Yes | 0 |
|  | 2018 | SDI | Facility level | 1050 | Yes | 0 |
|  | 2018 | SDI | Salary satisfaction | 1050 | No | - |
|  | 2018 | SDI | Overall satisfaction | 1050 | No | - |
|  | 2018 | SDI | Motivation | 1050 | No | - |
|  | 2018 | SDI | Unaurized absence | 1050 | Yes | 0 |
|  | 2018 | SDI | Outside employment | 1050 | No | - |
| Tajikistan | 2014 | RBF | Salary delay | 1085 | Yes | 0 |
|  | 2014 | RBF | Sex | 1085 | Yes | 0 |
|  | 2014 | RBF | Rural | 1085 | Yes | 0 |
|  | 2014 | RBF | Contract type | 1085 | No | - |
|  | 2014 | RBF | Age | 1085 | Yes | 0 |
|  | 2014 | RBF | Cadre | 1085 | Yes | 0 |
|  | 2014 | RBF | Facility level | 1085 | Yes | 0 |
|  | 2014 | RBF | Salary satisfaction | 1085 | Yes | 0 |
|  | 2014 | RBF | Overall satisfaction | 1085 | Yes | 0 |
|  | 2014 | RBF | Motivation | 1085 | Yes | 28 |
|  | 2014 | RBF | Unaurized absence | 1085 | Yes | 0 |
|  | 2014 | RBF | Outside employment | 1085 | Yes | 0 |
| Tanzania | 2014 | SDI | Salary delay | 1045 | Yes | 0 |
|  | 2014 | SDI | Sex | 1045 | Yes | 0 |
|  | 2014 | SDI | Rural | 1045 | Yes | 0 |
|  | 2014 | SDI | Contract type | 1045 | No | - |
|  | 2014 | SDI | Age | 1045 | Yes | 0 |
|  | 2014 | SDI | Cadre | 1045 | Yes | 0 |
|  | 2014 | SDI | Facility level | 1045 | Yes | 0 |
|  | 2014 | SDI | Salary satisfaction | 1045 | No | - |
|  | 2014 | SDI | Overall satisfaction | 1045 | No | - |
|  | 2014 | SDI | Motivation | 1045 | No | - |
|  | 2014 | SDI | Unaurized absence | 1045 | Yes | 0 |
|  | 2014 | SDI | Outside employment | 1045 | No | - |
|  | 2016 | SDI | Salary delay | 1158 | Yes | 0 |
|  | 2016 | SDI | Sex | 1158 | Yes | 0 |
|  | 2016 | SDI | Rural | 1158 | Yes | 0 |
|  | 2016 | SDI | Contract type | 1158 | No | - |
|  | 2016 | SDI | Age | 1158 | Yes | 1 |
|  | 2016 | SDI | Cadre | 1158 | Yes | 0 |
|  | 2016 | SDI | Facility level | 1158 | Yes | 0 |
|  | 2016 | SDI | Salary satisfaction | 1158 | No | - |
|  | 2016 | SDI | Overall satisfaction | 1158 | No | - |
|  | 2016 | SDI | Motivation | 1158 | No | - |
|  | 2016 | SDI | Unaurized absence | 1158 | Yes | 0 |
|  | 2016 | SDI | Outside employment | 1158 | No | - |
| Togo | 2013 | SDI | Salary delay | 439 | Yes | 0 |
|  | 2013 | SDI | Sex | 439 | Yes | 0 |
|  | 2013 | SDI | Rural | 439 | Yes | 0 |
|  | 2013 | SDI | Contract type | 439 | No | - |
|  | 2013 | SDI | Age | 439 | Yes | 0 |
|  | 2013 | SDI | Cadre | 439 | Yes | 0 |
|  | 2013 | SDI | Facility level | 439 | Yes | 0 |
|  | 2013 | SDI | Salary satisfaction | 439 | No | - |
|  | 2013 | SDI | Overall satisfaction | 439 | No | - |
|  | 2013 | SDI | Motivation | 439 | No | - |
|  | 2013 | SDI | Unaurized absence | 439 | Yes | 0 |
|  | 2013 | SDI | Outside employment | 439 | No | - |
| Uganda | 2013 | SDI | Salary delay | 416 | Yes | 0 |
|  | 2013 | SDI | Sex | 416 | Yes | 0 |
|  | 2013 | SDI | Rural | 416 | Yes | 0 |
|  | 2013 | SDI | Contract type | 416 | No | - |
|  | 2013 | SDI | Age | 416 | Yes | 1 |
|  | 2013 | SDI | Cadre | 416 | Yes | 0 |
|  | 2013 | SDI | Facility level | 416 | Yes | 0 |
|  | 2013 | SDI | Salary satisfaction | 416 | No | - |
|  | 2013 | SDI | Overall satisfaction | 416 | No | - |
|  | 2013 | SDI | Motivation | 416 | No | - |
|  | 2013 | SDI | Unaurized absence | 416 | Yes | 1 |
|  | 2013 | SDI | Outside employment | 416 | No | - |

Note: RBF = Results-Based Financing survey; SDI = Service Delivery Indicators survey. Collected? Indicates whether an indicator was included in the questionnaire for that wave (Yes if at least one non-missing observation exists; No if the item was not asked at all). % missing (when collected) is calculated as 100 ×(number of missing responses / N in the wave) among waves where the item was collected.

# **4 Robustness checks**

## **Figure A2. Salary Delay Patterns by Individual and Facility Characteristics: Sensitivity Analysis Using Missing Indicator Method for Covariates**

Note: MD = mean difference; pp = percentage points. The analysis includes 22,003 primary healthcare (PHC) workers from 16 LMICs. Estimated mean differences are from the linear probability models and are interpreted as the adjusted absolute difference (pp) in the probability of reporting a salary delay for each category relative to the indicated reference group, holding all other covariates and fixed effects constant. Models control for worker characteristics (contract type, gender, age, cadre), facility attributes (location and level), and fixed effects for country, year, and data source. Standard errors are clustered at the facility level. Missing covariate data were handled using the missing indicator method. Reference groups are indicated in parentheses.

## **Figure A3. Salary Delay and Health Worker Behaviors Association: Sensitivity Analysis Using Missing Indicator Method for Covariates**


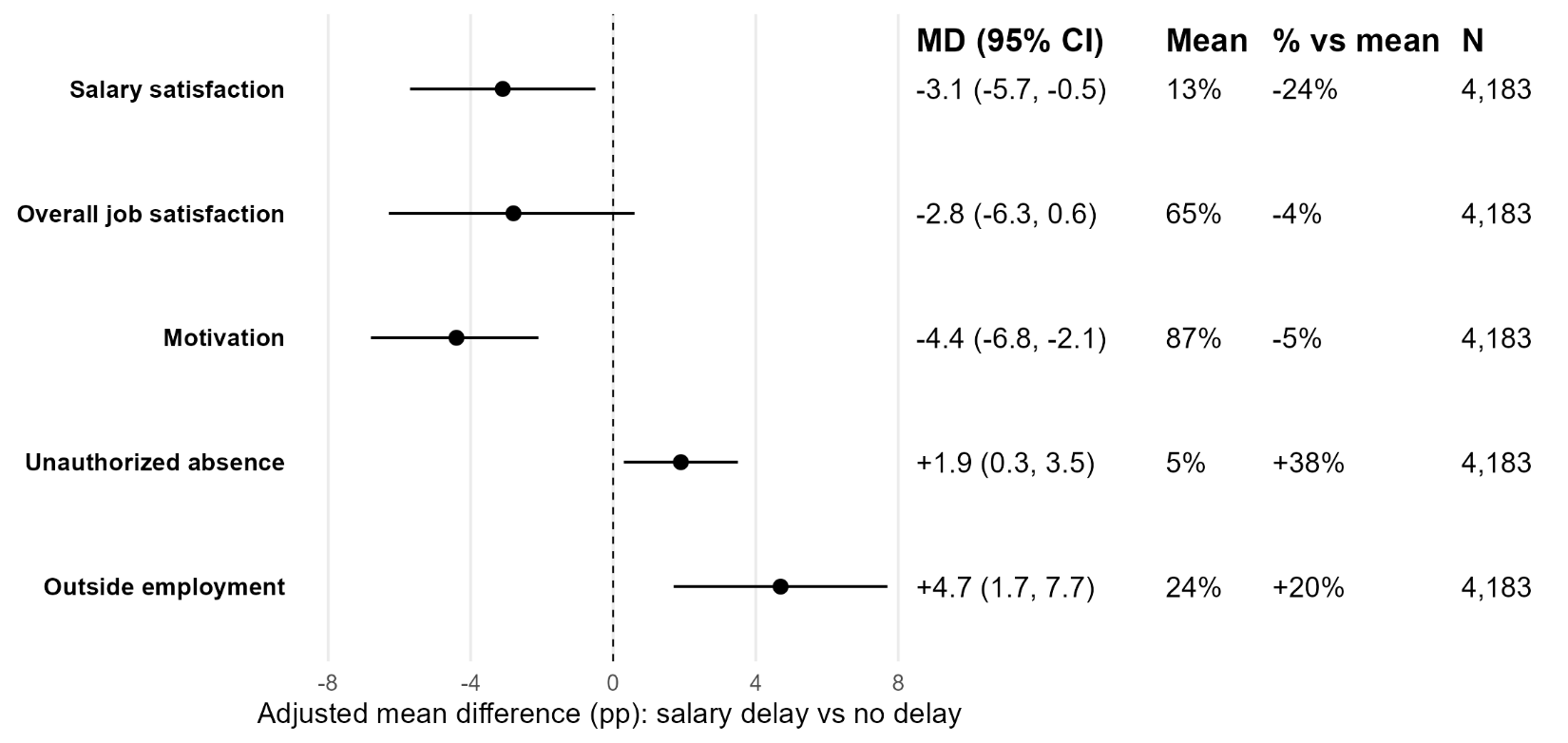


Note: MD = mean difference; pp = percentage points. The analysis includes 4,183 primary healthcare (PHC) workers with complete data on all outcomes from 5 RBF surveys to ensure consistent sample sizes across outcomes. Estimated mean differences are from the linear probability models and are interpreted as the adjusted absolute difference (pp) in the probability of each outcome (satisfied with salary, satisfied with job overall, feeling motivated, unauthorized absence, outside employment) for health workers reporting a salary delay versus those not, holding all other covariates and fixed effects constant. The “Mean” column gives the outcome mean in this analytic sample; “% vs mean” expresses the percent change relative to that mean. Models control for worker characteristics (contract type, gender, age, cadre), facility attributes (location and level), and fixed effects for country, year, and data source. Standard errors are clustered at the facility level. Missing covariate data were handled using the missing indicator method. Results are similar without the balanced-N restriction.

## **Table A8. Salary Delay and Health Worker Behaviors Association: Leave-One-Out Analysis Results**

| Country left out | N | **Probability of reporting high salary satisfaction** | **Probability of reporting high overall job satisfaction** | **Probability of reporting feeling motivated** | **Probability of unauthorized absenteeism** | **Probability of engaging in outside employment** |
| --- | --- | --- | --- | --- | --- | --- |
| Full Sample | 4,183 | -0.031 | -0.029 | -0.046 | 0.019 | 0.056 |
|  |  | [-0.057, -0.005] | [-0.063, 0.005] | [-0.070, -0.023] | [0.003, 0.035] | [0.025, 0.086] |
| CAR | 3,482 | -0.029 | -0.010 | -0.060 | 0.008 | 0.049 |
|  |  | [-0.062, 0.003] | [-0.048, 0.028] | [-0.088, -0.032] | [-0.004, 0.021] | [0.017, 0.082] |
| Cameroon | 3,907 | -0.036 | -0.032 | -0.043 | 0.024 | 0.054 |
|  |  | [-0.063, -0.010] | [-0.067, 0.003] | [-0.066, -0.019] | [0.007, 0.040] | [0.023, 0.085] |
| Lesotho | 4,128 | -0.032 | -0.024 | -0.046 | 0.019 | 0.057 |
|  |  | [-0.059, -0.006] | [-0.058, 0.010] | [-0.070, -0.023] | [0.003, 0.035] | [0.027, 0.088] |
| Nigeria | 1,814 | -0.034 | -0.024 | 0.019 | 0.028 | 0.063 |
|  |  | [-0.061, -0.006] | [-0.080, 0.032] | [-0.014, 0.052] | [-0.005, 0.061] | [0.013, 0.112] |
| Tajikistan | 3,401 | -0.031 | -0.056 | -0.062 | 0.021 | 0.038 |
|  |  | [-0.061, -0.002] | [-0.093, -0.019] | [-0.088, -0.037] | [0.002, 0.040] | [0.004, 0.071] |

Notes: The analysis includes 4,183 primary healthcare (PHC) workers with complete data on all outcomes from 5 RBF surveys to ensure consistent sample sizes across outcomes. Coefficients are on a 0-1 probability scale; multiply by 100 to express as percentage points. All models control for: worker characteristics (contract type, gender, age, cadre), facility attributes (location and level), and fixed effects for country, year, and data source. Standard errors are clustered at the facility level. Missing covariate values were handled by multiple imputations with chained equations.
